# Evidence that TNF-β (lymphotoxin α) can activate the inflammatory environment in human chondrocytes

**DOI:** 10.1186/ar4393

**Published:** 2013-11-28

**Authors:** Constanze Buhrmann, Parviz Shayan, Bharat B Aggarwal, Mehdi Shakibaei

**Affiliations:** 1 Musculoskeletal Research Group, Institute of Anatomy, Ludwig-Maximilian-University Munich, Pettenkoferstrasse 11, D-80336, Munich, Germany; 2 Investigating Institute of Molecular Biological System Transfer, Tehran 1417863171, Iran; 3 Cytokine Research Laboratory, Department of Experimental Therapeutics, The University of Texas, MD Anderson Cancer Center, Unit 143, 1515 Holcombe Boulevard, Houston, TX 77030, USA

## Abstract

**Introduction:**

Inflammatory cytokines play a key role in the pathogenesis of joint diseases such as rheumatoid arthritis (RA). Current therapies target mainly tumor necrosis factor α (TNF-α) as this has proven benefits. However, a large number of patients do not respond to or become resistant to anti-TNF-α therapy. While the role of TNF-α in RA is quite evident, the role of TNF-β, also called lymphotoxin-α (LT-α), is unclear. In this study we investigated whether TNF-β and its receptor play a role in chondrocytes in the inflammatory environment.

**Methods:**

An *in vitro* model of primary human chondrocytes was used to study TNF-β-mediated inflammatory signaling.

**Results:**

Cytokine-induced inflammation enhances TNF-β and TNF-β-receptor expression in primary human chondrocytes accompanied by the up-regulation of inflammatory (cyclooxygenase-2), matrix degrading (matrix metalloproteinase-9 and -13) and apoptotic (p53, cleaved caspase-3) signaling pathways, all known to be regulated by NF-κB. In contrast, anti-TNF-β, similar to the natural NF-κB inhibitor (curcumin, diferuloylmethane) or the knockdown of NF-κB by using antisense oligonucleotides (ASO), suppressed IL-1β-induced NF-κB activation and its translocation to the nucleus, and abolished the pro-inflammatory and apoptotic effects of IL-1β. This highlights, at least in part, the crucial role of NF-κB in TNF-β-induced-inflammation in cartilage, similar to that expected for TNF-α. Finally, the adhesiveness between TNF-β-expressing T-lymphocytes and the responding chondrocytes was significantly enhanced through a TNF-β-induced inflammatory microenvironment.

**Conclusions:**

These results suggest for the first time that TNF-β is involved in microenvironment inflammation in chondrocytes during RA parallel to TNF-α, resulting in the up-regulation of NF-κB signaling and activation of pro-inflammatory activity.

## Introduction

In 1984, one of our groups isolated two different cytokines, tumor necrosis factor α (TNF-α) and TNF-β, from macrophages and lymphocytes, respectively [1]. When we examined them for their receptors, we found that both cytokines bind to the same receptor [2]. Although the role of TNF-α in a wide variety of diseases, including rheumatoid arthritis (RA), is very well-documented, very little is known about TNF-β. Recent evidence suggests that TNF-β, alias lymphotoxin α (LT-α), another member of the TNF superfamily, may play a critical role in RA [3]. TNF-β shows 35% identity and 50% homology to TNF-α at amino acid sequences, making it the closest homolog to TNF-α and shows further structural similarity in tertiary and quaternary structure, indicating similar biological activity [2,4]. TNF-β is expressed by a variety of cells, including T cells, B cells and natural killer (NK) cells [5]. TNF-β can be secreted and, like TNF-α, binds with high affinity to TNF receptors 1 and 2 (TNFR-1 and TNFR-2) [4], and it is transiently expressed on the cell surfaces of activated B and T cells, where it forms a complex with LT-β as an LTα_1_β_2_ heterotrimer [6,7]. Recent evidence indicates that, for some physiological processes, TNF and LTαβ work together as components of an integrated signaling network that is defined in part by communal sharing of receptors and ligands [7].

RA is a chronic, systemic inflammatory autoimmune disease characterized by inflammation of the synovial joints [8]. Because of its persistent inflammatory environment, RA is accompanied by progressive joint degeneration, with pain and impairment of patients’ daily lives. Hallmarks of RA are enhanced proliferation of fibroblast-like synoviocytes (FLSs) accompanied by an increase in proinflammatory cytokines such as interleukin 1 (IL-1), IL-6 and TNF-α [9,10].

IL-1β is a well-studied mediator of cartilage destruction in osteoarthritis (OA) and RA. This mediating effect occurs by reducing chondrocyte proteoglycan synthesis, increasing synthesis of matrix metalloproteinases (MMPs) and releasing nitric oxide [11,12]. IL-6 is known to enhance inflammation through its action on T and B cells as well as monocytes and neutrophils, and it is involved in the activation of osteoclasts [13]. TNF-α, first discovered as an anticancer agent, is known to contribute to host defense against infection, but it is also involved in the pathogenesis of many diseases and plays a key role in stimulating the inflammatory response in RA, which leads to synovial proliferation as well as bone and cartilage destruction [5].

Current treatment regimens for RA often target a specific cytokine to suppress inflammatory processes [14-17]. Because TNF-α plays a major role in promoting RA, its inhibition has been used for the treatment of RA with very promising results [12,18]. Unfortunately, it has recently been shown that many patients do not, or only slightly, respond to anti-TNF-α therapy and that up to 50% of patients become resistant to TNF-α therapy after five years of treatment [19]. Furthermore, TNF-α therapy is implicated in the increased risk of serious infections and malignancies [20]. This set of problems demonstrates a necessity for additional efficacious and safe alternative therapies for RA.

Previous studies have indicated that TNF-β levels are elevated in the serum and synovial tissue of RA and OA patients [21-23]. A recent report demonstrated that TNF-β stimulates proliferation and inflammatory cascade signaling in FLSs, which is a trigger and initial starting point of RA [3]. Interestingly, in an *in vivo* collagen-induced arthritis mouse model, anti-TNF-β therapy dramatically improved the disease course comparably to anti-TNF-α therapy [24]. Recently, pateclizumab, a TNF-β antibody, has been evaluated in a phase I clinical trial with good tolerance [25].

For safe and effective therapeutic use, however, more detailed information on the role of TNF-β in RA is needed. This is especially true regarding the interaction between chondrocytes, as the major architects of functional joint tissue, and TNF-β, which should be evaluated in more detail to better understand the inflammatory and degradative processes of RA.

In the search for effective and safe RA therapies, natural compounds have also shown ability to suppress inflammatory processes through inhibition of TNF-α. Curcumin (diferuloylmethane), when isolated from the rhizomes of *Curcuma longa*, has strong anti-TNF-α activity and can further suppress TNF-α production through inhibition of nuclear factor κB (NF-κB) activation [26-28]. Also, in chondrocytes, curcumin inhibits inflammation and matrix degradation by suppressing NF-κB activation [28,29]. Antisense oligonucleotides (ASOs) have been reported to selectively downregulate the translation of subcellular target genes [30,31]. ASO-based chemicals have been developed as gene-silencing therapeutic agents for use in clinical trials, such as cancer studies [32,33].

Therefore, because an increasing body of evidence suggests that TNF-β may play a major role in an inflammatory joint environment, we decided to find out whether TNF-β and its receptor are expressed by primary human chondrocytes in an *in vitro* model of RA and to investigate the underlying signaling pathways.

## Methods

### Antibodies

Antibodies to active caspase 3, MMP-9 and MMP-13 were purchased from R&D Systems (Heidelberg, Germany). Anti-phospho-specific p65 (NF-κB/Ser536) was obtained from Cell Signaling Technology (Beverly, MA, USA). Anti-TNF-β, anti-TNF-α and anti-TNF-β receptor (anti-TNF-β-R) antibodies were obtained from eBioscience (Frankfurt, Germany). Monoclonal poly(ADP-ribose) polymerase (PARP) antibodies were purchased from Becton Dickinson (Heidelberg, Germany). Cyclooxygenase 2 (Cox-2) antibody was obtained from Cayman Chemical (Ann Arbor, MI, USA). Monoclonal anti-β-actin antibody was purchased from Sigma-Aldrich Chemie (Munich, Germany). Antibodies against p53 were obtained from Santa Cruz Biotechnology (Santa Cruz, CA, USA). Alkaline phosphatase–linked sheep anti-mouse and sheep anti-rabbit secondary antibodies for immunoblotting were purchased from EMD Millipore (Schwalbach, Germany). Secondary antibodies used for fluorescence labeling were purchased from Dianova (Hamburg, Germany). Gold particle–conjugated secondary antibodies were purchased from Amersham (Braunschweig, Germany). All antibodies were used at concentrations recommended by the manufacturers. TNF-β and polyclonal rabbit anti-TNF-β antibodies were used as described previously [34] and were kindly provided by BB Aggarwal (The University of Texas MD Anderson Cancer Center, Houston, TX, USA). Bacteria-derived recombinant human TNF and LT, both purified to homogeneity with a specific activity of 50 million U/mg, were kindly provided by Genentech (South San Francisco, CA, USA).

### Growth media, chemicals and cytokines

Cell culture growth medium consisting of Dulbecco’s modified Eagle’s medium/Ham’s F-12 (1:1), 10% fetal bovine serum (FBS), 1% partricin solution, 1% penicillin-streptomycin solution (10,000 IU/10,000 IU), 75 μg/ml ascorbic acid, 1% essential amino acids and 1% glutamine was obtained from Seromed (Munich, Germany). Epon was obtained from Plano (Marburg, Germany). Curcumin with a purity greater than 95% was purchased from Indsaff (Punjab, India) and dissolved in dimethyl sulfoxide, and a 5,000 μM stock was prepared and further diluted in cell culture medium. This commercial source of curcumin contains three major components: diferuloylmethane (the most abundant and active component of turmeric) (82%) and its derivatives demethoxycurcumin (15%) and bisdemethoxycurcumin (3%), together referred to as curcuminoids [35,36]. IL-1β was purchased from Acris Antibodies GmbH (Herold, Germany).

### Chondrocyte and T lymphocyte culture

Primary human chondrocytes (catalog no. 121 0211) were obtained from Provitro (Berlin, Germany). Briefly, during monolayer expansion, chondrocytes were seeded at a density of 300,000 cells/T75 cell culture flask and grown until 70% confluence was reached in cell culture growth medium (10% FBS) [37]. Chondrocytes were passaged up to two times, and passages 2 and 3 were used in the experiments. Chondrocytes were washed three times with serum-starved medium (containing only 3% FBS) and further incubated for 30 minutes with the same medium before initiation of treatment with TNF-β and/or inhibitors. Cartilage samples were derived from patients, who provided their full informed consent. Local ethics committee approval was provided by the Charité-University Medical School Berlin, Germany. A human T lymphocyte cell line (Jurkat cells) [38] was cultured in suspension with whole-cell culture growth medium.

### Time- and concentration-dependent experiments

To evaluate the effect of IL-1β on TNF-β expression in primary human chondrocytes, time- and dose-dependent experiments were carried out. Primary human chondrocytes in monolayer culture were either left untreated or treated with 10 ng/ml IL-1β for various durations (5, 10, 20, 40 or 60 minutes) or with various concentrations of IL-1β (0, 10, 15, 20 or 25 ng/ml) for 1 hour. Whole-cell extracts were prepared, and immunoblotting was carried out as described below.

### Antisense and Lipofectin-mediated transfection in chondrocytes

Transient transfection of primary human chondrocytes was performed as previously described [39]. Phosphorothioate-specific oligonucleotides (21-mer) in ASOs (sequence 5′-GAGATGCGCACTGTCCCTGGTC-3′) corresponding to NF-κB/p65 subunit mRNA and control 21-mer sense oligonucleotides (SOs) (sequence 5′-GACCAGGGACAGTGCGCATCTC-3′) used in the experiments were synthesized by Eurofins MWG Operon (Ebersberg, Germany). All ASOs and SOs were phosphorothioate-modified to protect them from the cell nucleases. Transfection was carried out by incubating the primary chondrocytes in monolayer culture for 24 hours with 0.5 μM ASO against NF-κB or SO control and 10 μl/ml Lipofectin transfection reagent (Invitrogen, Carlsbad, CA, USA) in serum-starved medium (3% FBS) before starting the respective experiments. All transfection experiments were carried out on 50% confluent monolayer cultures.

### Western blot analysis and immunoblotting

Whole-cell lysates or nuclear extracts used for Western blot analysis were prepared as previously described in detail [40,41]. Briefly, for whole-cell extracts, proteins were extracted with lysis buffer (50 mM Tris–HCl, pH 7.2, 150 mM NaCl, 1% (v/v) Triton X-100, 1 mM sodium orthovanadate, 50 mM sodium pyrophosphate, 100 mM sodium fluoride, 0.01% (v/v) aprotinin, 4 μg/ml pepstatin A, 10 μg/ml leupeptin, 1 mM phenylmethylsulfonyl fluoride) on ice for 30 minutes. For nuclear extracts, chondrocytes were trypsinized and washed twice in 1 ml of ice-cold phosphate-buffered saline (PBS). The cell pellet was resuspended in 400 μl of hypotonic lysis buffer containing protease inhibitors and incubated on ice for 15 minutes. Next, 12.5 μl of 10% Nonidet P-40 were added, and the cell suspension was vigorously mixed for 15 seconds. The extracts were centrifuged for 1.5 minutes at 14,000 × *g*. The supernatants (cytoplasmic extracts) were frozen at −80°C. Ice-cold nuclear extraction buffer (25 μl) was added to the pellets and incubated for 30 minutes with intermittent mixing. Extracts were centrifuged, and the supernatant (nuclear extracts) was transferred to the prechilled tubes for storage at −80°C. Total protein content was measured, samples were reduced with 2-mercaptoethanol and total protein concentrations were adjusted. After separation with SDS-PAGE under reducing conditions, the resultant was blotted onto a membrane using a Trans-Blot apparatus (Bio-Rad Laboratories, Hercules, CA, USA) and blocked for 2 hours in 5% (w/v) skimmed milk powder in PBS/0.1% Tween 20. Membranes were incubated overnight with the primary antibody directed against TNF-β, TNF-α, MMP-9 and −13, Cox-2, p53, cleaved caspase 3, NF-κB-p65, TNF-β-R, PARP and β-actin at a 1:1,000 dilution in blocking buffer at 4°C on a shaker, washed three times with blocking buffer, then incubated with the secondary antibody conjugated with alkaline phosphatase for 90 minutes at ambient temperature (AT). Membranes were washed three times in 0.1 M Tris (pH 9.5) containing 0.05 M MgCl_2_ and 0.1 M NaCl. Specific antigen–antibody complexes were detected using nitroblue tetrazolium and 5-bromo-4-chloro-3-indoylphosphate (*p*-toluidine salt; Pierce Biotechnology, Ulm, Germany) as substrates for alkaline phosphatase.

### Immunofluorescence analysis of TNF-β, TNF-β-R and NF-κB

The technique was performed as previously described in detail [29]. Briefly, primary human chondrocytes were cultured until 50% confluence was reached on four-well chamber slides or glass plates treated for the different experiments and fixated with methanol at AT for 10 minutes. Cells were rinsed three times with PBS and overlaid with bovine serum albumin (BSA) at AT. Primary antibodies (TNF-β, TNF-β-R and NF-κB (1:50) in PBS/BSA) incubation was performed overnight at 4°C in a humidified chamber, followed by incubation with rhodamine-coupled secondary antibodies (diluted 1:80 in PBS) for 1 hour at AT and washed again three times with aqua dest (that is, distilled) laboratory water. Counterstaining was performed with 4′,6-diamidino-2-phenylindole (DAPI; Sigma-Aldrich Chemie) to visualize cell nuclei. The slides were then covered with fluoromount mountant and examined under a fluorescence microscope (Leica Microsystems, Wetzlar, Germany).

### Immunoelectron microscopy (preembedding technique)

Detection of TNF-β-R expression on T lymphocytes (Jurkat cells) by immunoelectron microscopy was performed as previously described [42]. Jurkat cells were treated with anti-TNF-β-R for 10 minutes at a concentration of 1:20 in cell culture medium, followed by treatment with 10 nanometer goat anti-rabbit gold-conjugated secondary antibodies for an additional 10 minutes at AT (diluted 1:30 in growth medium). Cells were fixed with 2% glutaraldehyde for 5 minutes and postfixed in 1% OsO_4_ solution. After dehydration in a series of ethanol washes, pellets were embedded in Epon and ultrathin cuts were made on a Reichert-Jung Ultracut E microtome (Leica Microsystems) and contrasted with a mixture of 2% uranyl acetate/lead citrate. Sections were examined under a Zeiss 10 transmission electron microscope (Institute of Pharmacology, Berlin, Germany).

### Adhesion assay interaction of chondrocytes with T cells

Primary human chondrocytes were grown to subconfluence in a monolayer culture and either left unstimulated or stimulated with 1 or 10 ng/ml TNF-β for 12 hours, washed and then cocultured with T lymphocytes (Jurkat cells) (2 × 10^6^/ml) for 4 hours. Subsequently, nonadherent T cells were removed by gentle washing three times with PBS before paraformaldehyde fixation for evaluation by light microscopy.

## Results

The aim of the present study was to evaluate the potential inflammatory and apoptotic signaling role of TNF-β (alias LT-α) in an *in vitro* model of IL-1β-induced inflammatory environment, such as RA in primary human chondrocytes. Furthermore, we evaluated signaling interactions between TNF-β and the NF-κB pathway, as well as the potential beneficial role of curcumin in suppressing TNF-β-induced gene products that lead to inflammation, degradation and apoptosis. We purposely chose to stimulate the inflammatory reaction in the chondrocytes with IL-1β instead of TNF-α to avoid any possible cross-reactions between TNF-β and TNF-α signaling.

### IL-1β induces TNF-β and TNF-β-R expression in human chondrocytes

**Figure 1:**
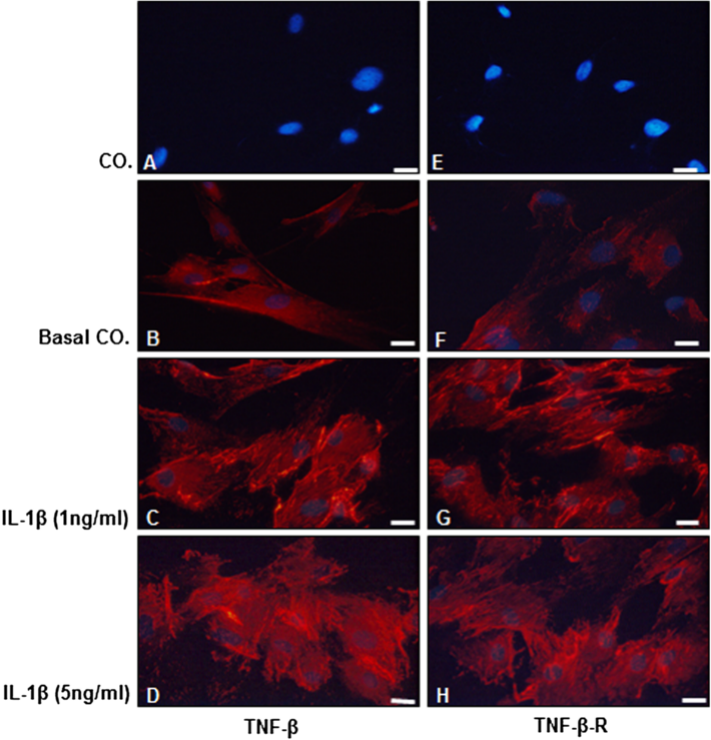
**Detection of TNF-β and TNF-β receptor expression in cultured human chondrocytes *in vitro *by immunofluorescence microscopy.** Primary human chondrocytes were either left untreated (Co. **(A)** and **(E)**: without primary antibody; basal-co.: **(B)**) and **(F)** with primary antibody) or treated for 1 hour with 1 ng/ml interleukin 1β (IL-1β) **(C)** and **(G)** or 5 ng/ml IL-1β **(D)** and **(H)**. Immunolabeling was performed with primary antibodies for tumor necrosis factor β (TNF-β) **(B)** through **(D)** and TNF-β receptor (TNF-β-R) **(E)** through **(H)**, followed by incubation with rhodamine-coupled secondary antibodies and counterstaining with 4′,6-diamidino-2-phenylindole to visualize cell nuclei. Images shown are representative of three different experiments. Original magnification, ×400; bar, 30 nm.

Because it is unknown whether healthy or inflamed chondrocytes express TNF-β or TNF-β-R, we evaluated whether IL-1β stimulates chondrocytes to express TNF-β and TNF-β-R. Primary human chondrocytes in monolayer cultures were either left untreated (Figure 1A and 1E: control (CO.), without primary antibody; Figure 1B and 1F: basal control (Basal CO.), with primary antibody) or stimulated with 1 ng/ml IL-1β (Figure 1C and 1G) or 5 ng/ml IL-1β (Figure 1D and 1H) for 1 hour and subjected to immunolabeling with anti-TNF-β or anti-TNF-β-R and rhodamine-coupled secondary antibodies. DAPI counterstaining was performed to visualize cell nuclei. We found that untreated chondrocytes had low basal expression of TNF-β (Figure 1B) and TNF-β-R (Figure 1F). However, inflammatory stimulation with IL-1β markedly induced TNF-β and TNF-β-R expression in chondrocytes at both at 1 and 5 ng/ml concentrations (Figure 1C, 1D, 1G and 1H).

### IL-1β induces TNF-β protein in a dose- and time-dependent manner in human chondrocytes

**Figure 2:**
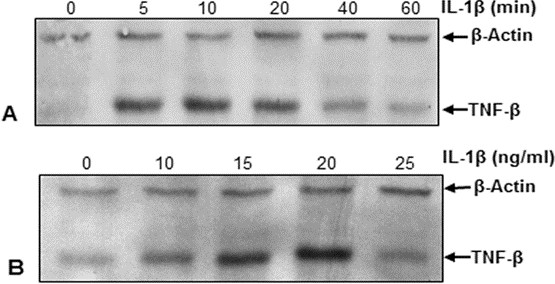
**IL-1β induces TNF-β protein expression in chondrocytes in a time- and dose-dependent manner.** Primary human chondrocytes were either treated with 10 ng/ml interleukin 1β (IL-1β) for the indicated time points **(A)** or treated with various concentrations of IL-1β for 1 hour **(B)**. Whole-cell lysates were prepared and analyzed by Western blotting with antibodies against tumor necrosis factor β (TNF-β). The results shown are representative of three independent experiments. Housekeeping protein β-actin served as a loading control in all experiments.

We next examined whether the observed IL-1β-induced expression of TNF-β in primary human chondrocytes was time- and dose-dependent. Primary human chondrocytes were either treated with 10 ng/ml IL-1β for the indicated time points (Figure 2A) or treated with various concentrations of IL-1β for 1 hour (Figure 2B). Immunoblotting of whole-cell extracts shows a time-dependent increase in TNF-β expression in chondrocytes during IL-1β treatment, reaching a maximum production level of TNF-β after 10 minutes (Figure 2A). Moreover, we observed a clear, dose-dependent correlation between increasing dosages of IL-1β (with the strongest effect occurring at 20 ng/ml) and markedly increasing production of TNF-β (Figure 2B).

### Anti-TNF-β, ASO against NF-κB, curcumin reduce IL-1β-induced TNF-β-, TNF-α production and NF-κB-regulated proinflammatory and apoptotic proteins

**Figure 3:**
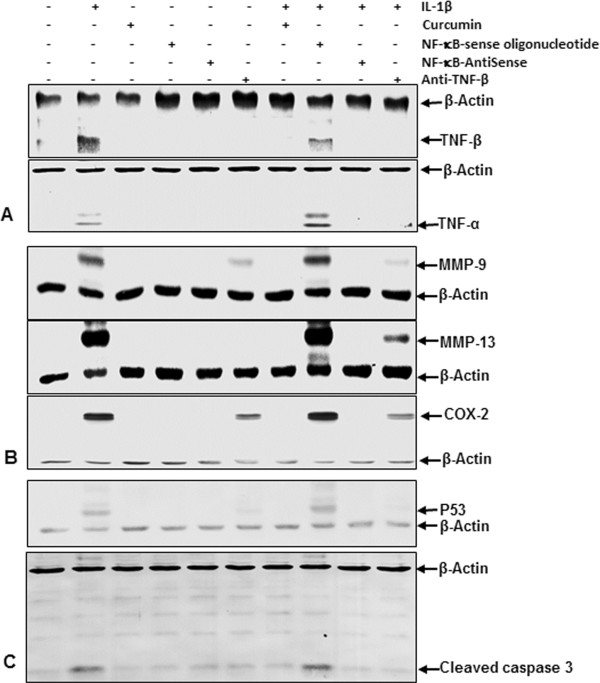
**Effects of antisense oligonucleotides against NF-κB, curcumin and anti-TNF-β on IL-1β-induced TNF-β expression, TNF-α expression and NF-κB-regulated proinflammatory and apoptotic proteins in human chondrocytes.** Serum-starved primary human chondrocytes were (1) either left untreated or treated with 10 ng/ml interleukin 1β (IL-1β), 5 μM curcumin, 0.5 μM sense oligonucleotide (SO) control or antisense oligonucleotides (ASOs) against NF-κB in the presence of Lipofectin transfection reagent (10 μl/ml) or 10 ng/ml anti-tumor necrosis factor β (anti-TNF-β) alone for 24 hours; or (2) cells were pretreated with 10 ng/ml IL-1β for 1 hour, followed by cotreatment with 5 μM curcumin, 0.5 μM SO control or ASO against nuclear factor κB (NF-κB) in the presence of 10 μl/ml Lipofectin transfection reagent or 10 ng/ml anti-TNF-β for 24 hours. Whole-cell extracts were fractionated on SDS-PAGE gels and analyzed by Western blotting with antibodies against TNF-β and TNF-α **(A)**, matrix metalloproteinase 9 (MMP-9), MMP-13 and cyclooxygenase 2 (COX-2) **(B)** and p53 and cleaved caspase 3 **(C)**. Western blots shown are representative of three independent experiments. Housekeeping protein β-actin served as a loading control in all experiments.

Because we could demonstrate that IL-1β induces TNF-β expression in chondrocytes, we next investigated whether this expression is mediated through the NF-κB signaling pathway (Figure 3). Whole-cell extracts were evaluated by Western blot analysis after (1) primary human chondrocytes were either left untreated or treated with 10 ng/ml IL-1β, 5 μM curcumin, 0.5 μM SO control or ASOs against NF-κB in the presence of either 10 μl/ml Lipofectin transfection reagent or 10 ng/ml anti-TNF-β for 24 hours; or (2) cells were pretreated with 10 ng/ml IL-1β for 1 hour followed by cotreatment with 5 μM curcumin, 0.5 μM SO control or ASOs against NF-κB in the presence of 10 μl/ml Lipofectin transfection reagent or 10 ng/ml anti-TNF-β for another 24 hours.

#### IL-1β-induced production of TNF-β and TNF-α is specifically inhibited by curcumin, ASOs against NF-κB and anti-TNF-β

Western blot analysis of whole-cell extracts showed specific inhibition of IL-1β-induced TNF-β as well as TNF-α production in the presence of ASOs against NF-κB, demonstrating that the NF-κB signaling pathway plays a significant role in the production of TNF-β and TNF-α in chondrocytes (Figure 3A). An SO control had no effect on TNF-β or TNF-α protein expression. Treatment with the natural NF-κB inhibitor curcumin also completely inhibited TNF-β and TNF-α production in chondrocytes. Interestingly, treatment with anti-TNF-β inhibited not only TNF-β but also TNF-α production, indicating a possible negative feedback mechanism of TNF-β, which downregulates TNF-α production.

#### IL-1β-induced, NF-κB-regulated, proinflammatory and matrix-degrading proteins are inhibited by curcumin, ASOs against NF-κB and anti-TNF-β

As IL-1β is well-known to upregulate proinflammatory and matrix-degrading proteins in chondrocytes, all of which are known to be regulated by NF-κB [43], we next examined the expression of MMP-9, MMP-13 and Cox-2. As demonstrated by immunoblotting of total cell lysates, IL-1β-induced TNF-β production significantly enhances MMP-9, MMP-13 and Cox-2 production and is completely blocked in the presence of ASOs against NF-κB or curcumin (Figure 3B). An SO control had no effect on MMP-9, MMP-13 and Cox-2 production. Treatment with anti-TNF-β only partly blocks MMP-9, MMP-13 and Cox-2 production, indicating that IL-1β-induced inflammatory and matrix-degrading signaling is mediated only in part by TNF-β signaling through the NF-κB pathway.

#### IL-1β-induced NF-κB-dependent expression of apoptotic proteins is specifically downregulated by curcumin, ASOs against NF-κB and anti-TNF-β

It is well-known that the NF-κB signaling pathway is involved in cellular apoptosis in chondrocytes [43]. Therefore, we next investigated whether blocking TNF-β inhibits NF-κB-dependent apoptosis. Equal amounts of total proteins were separated by SDS-PAGE and analyzed by immunoblotting with antibodies raised against cleaved caspase 3 and p53 (Figure 3C). Transfection with ASOs against NF-κB or treatment with the natural NF-κB inhibitor curcumin completely inhibited cleavage of caspase 3 and p53 production. An SO control had no effect on the cleavage of caspase 3 and p53 expression. Treatment with anti-TNF-β also completely blocked cleavage of caspase 3 and p53 production, indicating that TNF-β might be involved in inducing apoptosis in chondrocytes through activation of caspase 3 and p53.

### IL-1β-induced phosphorylation and translocation of NF-κB-p65 is inhibited by anti-TNF-β, ASOs against NF-κB and curcumin

**Figure 4:**
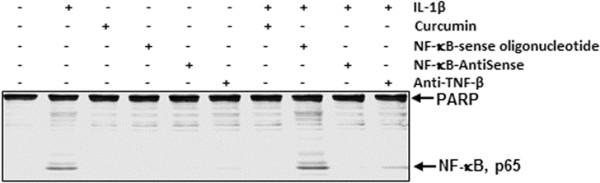
**Effects of antisense oligonucleotide against NF-κB, curcumin and anti-TNF-β on IL-1β-induced phosphorylation and translocation of NF-κΒ-p65 in nuclear extracts in human chondrocytes.** (1) Serum-starved primary human chondrocytes were either left untreated or were treated with 10 ng/ml interleukin 1β (IL-1β), 5 μM curcumin, 0.5 μM sense oligonucleotide (SO) control or antisense oligonucleotide (ASO) against nuclear factor κB (NF-κB) in the presence of 10 μl/ml Lipofectin transfection reagent or 10 ng/ml anti-tumor necrosis factor β (anti-TNF-β) alone for 24 hours; or (2) cells were pretreated with 10 ng/ml IL-1β for 1 hour, followed by cotreatment with 5 μM curcumin, 0.5 μM SO control or ASO against NF-κB in the presence of 10 μl/ml Lipofectin transfection reagent or 10 ng/ml anti-TNF-β for 24 hours. Nuclear cell fractions were prepared and examined by Western blot analysis using antibodies against phospho-specific p65 and nuclear protein poly(ADP-ribose) polymerase as a loading control. Western blots shown are representative of three independent experiments.

It has been reported that translocation of NF-κB to the nucleus is necessary for the regulation of gene expression by NF-κB. The translocation of activated NF-κB is preceded by phosphorylation of the p65 subunit of NF-κB [44,45]. Therefore, we next investigated whether IL-1β-induced phosphorylation and translocation of NF-κB is influenced by TNF-β. (1) Primary human chondrocytes were either left untreated or treated with 10 ng/ml IL-1β, 5 μM curcumin, 0.5 μM SO control or ASOs against NF-κB in the presence of 10 μl/ml Lipofectin transfection reagent or 10 ng/ml anti-TNF-β for 24 hours alone; or (2) cells were pretreated with 10 ng/ml IL-1β for 1 hour followed by cotreatment with 5 μM curcumin, 0.5 μM SO control or ASOs against NF-κB in the presence of 10 μl/ml Lipofectin transfection reagent or 10 ng/ml anti-TNF-β for another 24 hours (Figure 4). Western blot analysis of nuclear extracts showed that treatment with IL-1β alone or in combination with an SO control significantly induced NF-κB activation through enhanced p65 translocation to the nucleus. However, culturing with ASOs against NF-κB or the natural NF-κB inhibitor curcumin completely blocked IL-1β-induced NF-κB-p65 translocation to the nucleus. Furthermore, the SO control had no inhibitory effect on IL-1β-induced NF-κB-p65 translocation to the nucleus. Treatment with anti-TNF-β did not completely block IL-1β-induced NF-κB-p65 translocation to the nucleus, but resulted in a significant decrease in translocated NF-κB-p65 to the nuclear extract (Figure 4).

### Specific ASOs against NF-κB and/or curcumin block IL-1β-induced nuclear translocation of p65 and suppresses TNF-β production

**Figure 5:**
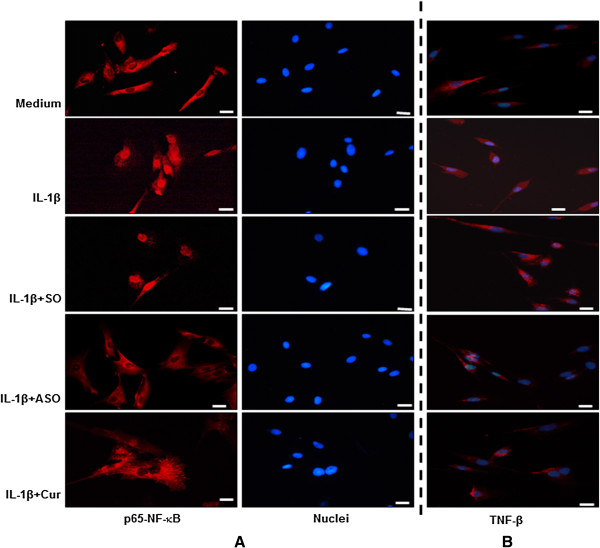
**NF-κB-p65 localization and TNF-β expression in human chondrocytes after treatment with IL-1β and/or specific antisense oligonucleotides against NF-κB or with curcumin.** (1) Serum-starved primary human chondrocytes were either left untreated (medium) or treated for 1 hour with 10 ng/ml interleukin 1β (IL-1β), or (2) cells were pretreated with 10 ng/ml IL-1β for 1 hour followed by cotreatment with 5 μM curcumin (Cur) for 4 hours, transfected with 0.5 μM sense oligonucleotide (SO) control or antisense oligonucleotide (ASO) against NF-κB in the presence of 10 μl/ml Lipofectin transfection reagent for 24 hours. For immunolabeling, cells were incubated with primary antibodies against nuclear factor κB (NF-κB)-p65 **(A)** or tumor necrosis factor β (TNF-β) **(B)**, followed by incubation with rhodamine-coupled secondary antibodies and counterstaining with 4′,6-diamidino-2-phenylindole to visualize cell nuclei. Images shown are representative of three different experiments. Original magnification, ×400; bar, 30 nm.

On the basis of Western blotting results (Figure 4), and to examine whether inhibition of NF-κB nuclear translocation influences IL-1β-induced TNF-β production in chondrocytes, we performed immunocytochemical analysis. Primary human chondrocytes in monolayer culture (1) were either left untreated or treated for 1 hour with 10 ng/ml IL-1β; (2) were treated with 0.5 μM SO control or ASO against NF-κB in the presence of Lipofectin transfection reagent for 24 hours; or (3) were pretreated with the natural NF-κB inhibitor curcumin (5 μM) for 4 hours followed by cotreatment with 10 ng/ml IL-1β for 1 hour. Immunolabeling was then performed with primary antibodies to NF-κB-p65 (Figure 5A) and TNF-β (Figure 5B), followed by incubation with rhodamine-coupled secondary antibodies. Counterstaining was performed with DAPI to visualize the cell nuclei. Treatment with IL-1β alone or in combination with SO control strongly induced NF-κB activation through enhanced p65 translocation to the nucleus. In contrast, ASO against NF-κB and curcumin treatment completely blocked IL-1β-induced p65 nuclear translocation (Figure 5A), indicating that SO control had no inhibitory effect on p65 translocation to the nucleus.

Treatment with IL-1β alone or IL-1β in combination with SO control strongly induces TNF-β production in chondrocytes (Figure 5B). Interestingly, TNF-β production is markedly suppressed in cultures treated with IL-1β and ASO against NF-κB or IL-1β and curcumin, indicating that, indeed, the activation of NF-κB through p65 nuclear translocation plays an essential role in TNF-β production in chondrocytes (Figure 5B).

### IL-1β induces TNF-β-R expression in T lymphocytes

**Figure 6:**
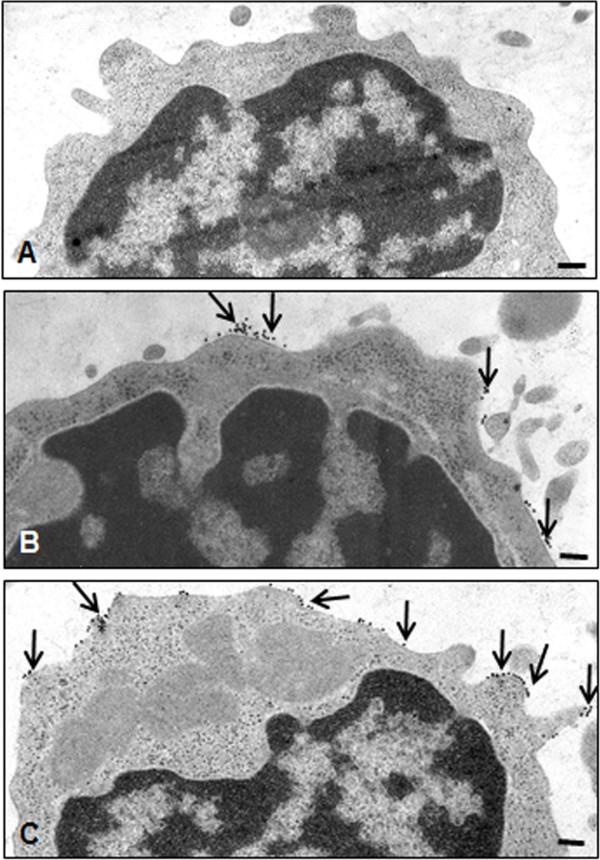
**Immunoelectron microscopic images showing tumor necrosis factor β receptor in T lymphocytes by immunogold labeling.** T lymphocytes were either left untreated without primary antibody **(A)** or with primary antibody **(B)** or treated with 5 ng/ml interleukin 1β (IL-1β) **(C)**. Immunogold labeling (arrows) is detected at the plasma membrane of lymphocytes with antibodies against tumor necrosis factor β receptor. Images shown are representative of three different experiments. Original magnification, ×25,000; bar, 0.25 μm.

TNF-β and TNF-β-R are expressed by a variety of cells including T cells, B cells and NK cells [5]. We performed immunoelectron microscopy [42] to investigate whether IL-1β treatment enhances TNF-β-R expression in T lymphocytes. T lymphocytes (Jurkat cells) were either left untreated (Figure 6B) or treated with 5 ng/ml IL-1β for 1 hour (Figure 6C). Immunoelectron microscopic images in Figure 6 show low basal expression of TNF-β-R in T lymphocytes (Figure 6B). Inflammatory stimulation with IL-1β treatment stimulated T lymphocytes to markedly enhance TNF-β-R production (Figure 6C).

### TNF-β induces TNF-β and TNF-β-R expression on surface of chondrocytes and enhances adhesiveness to T lymphocytes

**Figure 7:**
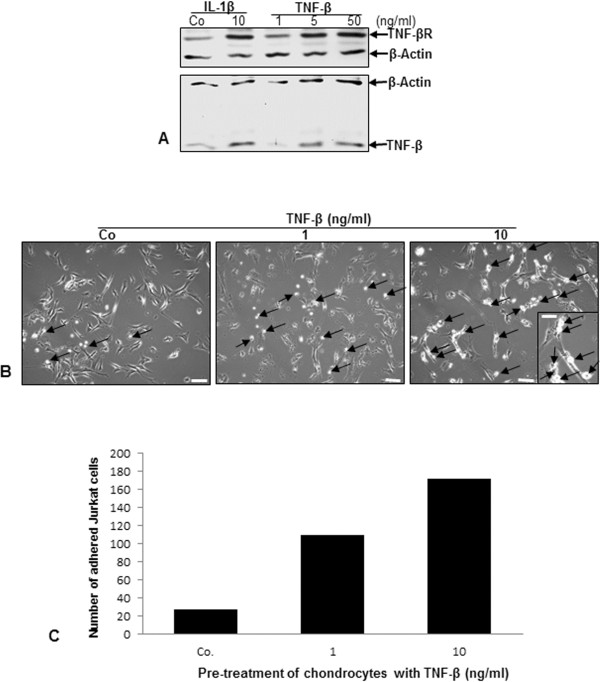
**Upregulation of TNF-β and TNF-β receptor expression in chondrocytes and adhesiveness of T lymphocytes to chondrocytes by TNF-β. (A)** Serum-starved chondrocytes were grown to subconfluence in monolayer culture and either left untreated (Co) or treated with 10 ng/ml interleukin 1β (IL-1β) or with 1, 5 or 50 ng/ml tumor necrosis factor β (TNF-β) for 12 hours. Whole-cell lysates were prepared, and samples were examined by Western blot analysis with antibodies against TNF-β and TNF-β receptor (TNF-βR). Western blots shown are representative of three independent experiments. The housekeeping protein β-actin served as a loading control. **(B)** Chondrocytes were grown to subconfluence in monolayer culture (spindle-shaped to elongated cells) and were either left untreated (Co) or treated with 1 or 10 ng/ml TNF-β for 12 hours, washed and cocultured with Jurkat cells (round to ovoid cells) for 4 hours. After being washed with phosphate-buffered saline, adhesion of T lymphocytes (arrows) to chondrocytes was evaluated by light microscopy. Original magnification, ×100; bar, 30 nm. Inset: original magnification, ×400; bar, 3 nm. **(C)** The number of Jurkat cells adherent to chondrocytes was estimated and quantified by counting ten microscopic fields per culture. Chondrocytes were either left unstimulated (Co) or stimulated with 1 or 10 ng/ml TNF-β for 12 hours.

As TNF-α is well-known to stimulate its own production [46], we next investigated whether, similarly, TNF-β stimulates itself in chondrocytes by enhancing TNF-β and TNF-β-R production. Primary human chondrocytes were grown to subconfluence in monolayer culture and either left untreated or treated with 10 ng/ml IL-1β or with 1, 5 or 50 ng/ml TNF-β for 12 hours. Western blot analysis demonstrated a marked dose-dependent increase in TNF-β and TNF-β-R production in chondrocytes as a result of TNF-β treatment (Figure 7A). Inflammation in RA is mediated through recruitment of lymphocytes to the site of inflammation [47]. Therefore, we next performed an adhesion assay to investigate whether TNF-β-induced inflammation in chondrocytes actively attracts lymphocytes to enhance inflammation in the RA microenvironment. Primary human chondrocytes were grown to subconfluence in monolayer culture and treated with 0, 1 or 10 ng/ml TNF-β for 12 hours. Jurkat cells (2 × 10^6^/ml) were cocultured with chondrocytes and incubated for 4 hours, and the number of T lymphocytes adherent to the surface of chondrocytes was quantified by light microscopy. Treatment with TNF-β did not morphologically change the chondrocytes. However, stimulation of chondrocytes with increased dosages of TNF-β resulted in an almost twofold increase in the adherence of T lymphocytes (Figure 7B and 7C), demonstrating the active role that chondrocytes may play during RA in retaining lymphocytes in the joint, thus supporting an inflammatory milieu and stimulating ongoing inflammation.

## Discussion

**Figure 8:**
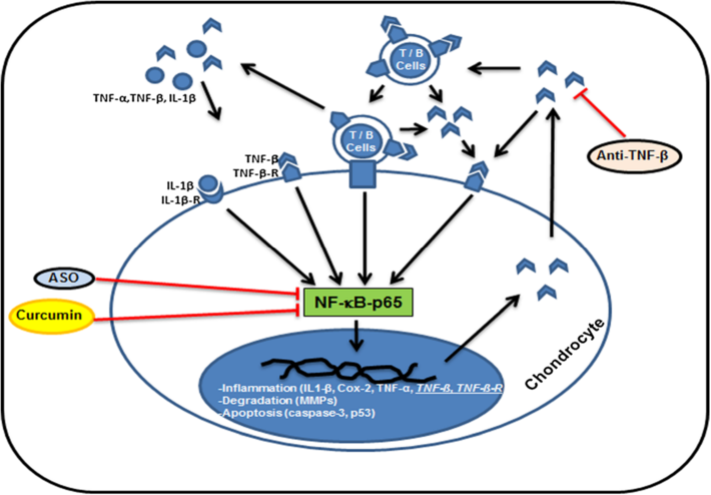
**Schematic showing TNF-β-induced proinflammatory and apoptotic signaling pathways in primary human chondrocytes in rheumatoid arthritis.** Proinflammatory cytokines (interleukin 1β (IL-1β), tumor necrosis factor α (TNF-α) and TNF-β) reach the synovial fluid through the bloodstream and can directly induce inflammation in chondrocytes, which leads to expression and production of cytokine receptors such as IL-1β and TNF-β receptors in the chondrocytes. TNF-β-induced inflammatory signaling in chondrocytes is further enhanced by upregulation of TNF-β production in T and B cells, by direct interaction of T and B cells with the chondrocytes and by autostimulation of the chondrocytes themselves. Downstream inflammation signaling of TNF-β in chondrocytes involves nuclear factor κB (NF-κB), leading to the production of proinflammatory mediators (IL-1β, TNF-α, TNF-β and cyclooxygenase 2 (Cox-2)), cartilage matrix degradation (matrix metalloproteinase 9 (MMP-9) and MMP-13) and apoptotic cascades (cleavage of caspase 3 and p53). The natural NF-κB inhibitor curcumin, antisense oligonucleotide against NF-κB or anti-TNF-β treatment can disrupt the TNF-β-induced inflammatory cycle through inhibition of the NF-κB signaling cascade, thus offering a promising therapeutic approach for inhibiting TNF-β-induced inflammatory environment in rheumatoid arthritis.

The goal of this study was to evaluate the potential role of TNF-β and its receptor in primary human chondrocytes and the underlying signaling mechanisms involved in an *in vitro* model of an inflammatory environment (Figure 8). The results presented herein led us to the following conclusions: (1) The proinflammatory cytokine IL-1β induced TNF-β and TNF-β-R expression in chondrocytes. (2) Anti-TNF-β suppressed IL-1β-induced TNF-β and TNF-α expression, which was associated with the downregulation of NF-κB-dependent, proinflammatory proteins (MMPs and Cox-2) and apoptotic proteins (p53 and cleavage of caspase 3). (3) Knockdown of NF-κB by mRNA downregulated the expression and activation of IL-1β-induced TNF-β in human chondrocytes. (4) IL-1β-induced NF-κB-p65 phosphorylation, and its translocation to the nucleus was inhibited by anti-TNF-β, curcumin or specific ASOs against NF-κB. (5) The adhesiveness between TNF-β-expressing T lymphocytes and the responding chondrocytes in cocultures was significantly enhanced in a TNF-β-induced inflammatory microenvironment.

It is known that TNF-α plays a major multifunctional role in the pathogenesis of RA and that blocking this proinflammatory cytokine is an effective therapeutic target for treating patients with RA. However, a significant number of RA patients do not respond adequately to this treatment, become resistant to anti-TNF-α therapies or experience significant adverse side effects [19,20,48], indicating the participation of other cytokines in the pathogenesis of RA. Therefore, additional development of new, more selective therapeutic targets is needed to better control inflammation and joint destruction in RA. Furthermore, several lines of evidence suggest that, in addition to well-studied cytokines in RA, such as IL-1β, IL-6 and TNF-α, other proinflammatory cytokines may be involved [49,50]. Attention has turned especially toward TNF-β (alias LT-α), another member of the TNF superfamily [3] and the closest homolog to TNF-α. Evidence indicates that TNF-β is associated with autoimmune and inflammatory diseases, and elevated synovial TNF-β levels have been found in RA patients [21,47,51,52]. It has previously been shown that *in vitro* TNF-β induces FLSs to adopt an aggressive phenotype, proliferate and actively produce proinflammatory cytokines [3]. In addition, anti-TNF-β treatment was found to significantly improve clinical symptoms in a collagen-induced RA mouse model. Indeed, pateclizumab, a novel monoclonal antibody targeting TNF-β, has recently been evaluated in RA patients [25]. A case report in the literature described a patient who did not respond to anti-TNF-α therapy with infliximab, but responded well to etanercept, a TNFR-2 crystallizable fragment fusion protein [21]. Because TNFR-2 is a receptor not only for TNF-α but also for TNF-β, this further indicates that TNF-β may play a larger role in inducing and/or enhancing RA than previously believed. Despite the advances in understanding the functional role of TNF-α in RA, little is known about the participation of TNF-β in this disease and remains to be elucidated.

We found that untreated primary human chondrocytes had very low expression of TNF-β, but that TNF-β expression in chondrocytes could be significantly induced time- and dose-dependently by treatment with the proinflammatory cytokine IL-1β. Furthermore, we have shown that anti-TNF-β treatment completely inhibited IL-1β-induced TNF-β and TNF-α production in chondrocytes, indicating a possible negative feedback mechanism in which, at least in part, TNF-α expression is regulated by TNF-β. Interestingly, it has been previously reported that proinflammatory cytokines TNF-α and TNF-β are strong stimulators of IL-1β [53], indicating that these cytokines are able to induce each other. These observations suggest that TNF-β, like IL-1β, may be actively involved in the inflammation and destruction that occurs in rheumatoid joints. These considerations led us to examine whether IL-1β induces the expression of TNF-β in chondrocytes *in vitro*.

Several lines of evidence suggest that proinflammatory cytokines (IL-1β and TNF-α) activate the ubiquitous transcription factor NF-κB, which leads to further proinflammatory cytokine expression and thus to further tissue degradation. Activated NF-κB, also in chondrocytes, is known to be involved in the regulation of several genes, including by infection, adhesion, cell-cycle, apoptosis, survival and inflammatory processes, by activating IL-1β, TNF-α, IL-6, Cox-2 and MMPs [2,5,37,46,54-58]. TNF-β has been shown to possess very early similar but less potent inflammatory potential than TNF-α [59]; however, the signaling transduction pathway of TNF-β has not been explored in chondrocytes. Furthermore, the natural polyphenol compound curcumin has been shown to have strong anti-proinflammatory activity and to be a natural NF-κB inhibitor in various cells, including chondrocytes [26-29]. NF-κB is part of an inactive cytoplasmic heterotrimer complex in association with inhibitor of NF-κB, α subunit (IκBα). For activation of gene transcription, following phosphorylation and ubiquitination, the p65 and p50 subunits dissociate from the IκBα complex and translocate to the nucleus to bind to NF-κB recognition sites in the promoter regions [45].

A possible mechanism underlying the inhibition of inducible NF-κB by curcumin (a natural NF-κB inhibitor) could be its capacity to inhibit the TNF-β signaling pathways in chondrocytes. Furthermore, our results demonstrate that anti-TNF-β inhibits IL-1β-induced NF-κB activation and its translocation to the nucleus in chondrocytes, demonstrating that TNF-β stimulates activation of NF-κB in chondrocytes upstream of IκBα dissociation, similarly to the signaling mechanism observed for TNF-α or lipopolysaccharide in chondrocytes [28,60]. These observations suggest that the TNF-β pathway may be involved in IL-1β-induced NF-κB signaling. We also found that anti-TNF-β, similarly to curcumin or specific ASOs against NF-κB, inhibits the proapoptotic protein caspase 3 and p53, the matrix-degrading MMPs, as well as the inflammatory enzyme Cox-2, that are regulated by NF-κB, suggesting the involvement of NF-κB in the regulation of TNF-β-induced proapoptotic and degradative pathways in cartilage. The inhibition of these proinflammatory cytokine-specific proteins by ASOs against NF-κB and anti-TNF-β underlines the similar characteristics that TNF-α and TNF-β exhibit in chondrocytes. The observation that TNF-α, a major cytokine present in the rheumatoid joints, stimulates the NF-κB signaling pathway has led to the approval of several TNF-α blockers in RA [5], and similar considerations might be given to the use of TNF-β in the future.

We next investigated whether inhibition of NF-κB activation and its nuclear translocation influenced IL-1β-induced TNF-β production in chondrocytes. Previously, our group has shown that, in the human myelomonoblastic leukemic cell line ML-1a, both TNF-α and TNF-β induce activation of NF-κB by signaling through TNF receptor subunits P80 and P60 [61]. In this study, we have shown that knockdown of NF-κB by ASOs downregulated the expression and activation of IL-1β-induced TNF-β in human chondrocytes. These results suggest that downregulation of TNF-β by NF-κB mRNA interference abrogates its stimulated effects on NF-κB and the proteins involved in inflammation and apoptosis, thus highlighting the importance of the TNF-β/NF-κB pathway during RA. To the best of our knowledge, this study is the first investigation that describes the effects of ASOs against NF-κB on TNF-β signaling pathways in human chondrocytes.

We observed that human chondrocytes express TNF-β receptors; therefore, further experiments were performed to determine their responsiveness to stimulation with TNF-β. In the present study, we have demonstrated that TNF-β treatment markedly induced its own expression in chondrocytes in a manner comparable to that of TNF-α [46]. We have further shown that creating an inflammatory microenvironment in chondrocytes through TNF-β stimulation enhanced the adhesiveness of T lymphocytes to chondrocytes. Moreover, it is known that NF-κB also regulates the expression of several genes that encode adhesion molecules such as intercellular adhesion molecule 1, vascular cell adhesion molecule 1 and E-selectin [62] and that these in turn are responsible for the recruitment of neutrophils, eosinophils and T lymphocytes from the circulation to the site of inflammation in chronic inflammatory diseases. It is further known that in RA FLSs actively bind T and B lymphocytes through LTα_1_β_2_ and induce the production of proinflammatory chemokines [63]. These results therefore suggest a new role for chondrocytes during RA in the retention of lymphocytes in the joint in supporting an inflammatory milieu and in stimulating ongoing inflammation mediated by TNF-β/NF-κB signaling.

## Conclusions

Our study demonstrates for the first time that TNF-β is an important regulator in activation of the NF-κB signaling pathway and its regulated proteins involved in inflammation, matrix degradation and apoptosis in human chondrocytes *in vitro*. We have further shown that TNF-β treatment stimulated chondrocytes and made them attractive for adhesion of T lymphocytes, indicating a connection between TNF-β signaling in chondrocytes and the recruitment of T lymphocytes for the upkeep of microenvironment inflammation in the rheumatoid joint. These results demonstrate that, during inflammation in chondrocytes, TNF-β signals similarly to TNF-α. As many patients only weakly respond to anti-TNF-α therapy, or even become resistant to it [19], blocking of TNF-β in combination with other TNF family members may be useful in the development of future new therapies for the treatment of inflammatory joint diseases such as RA.

## Abbreviations

ASO: Antisense oligonucleotide; AT: Ambient temperature; BSA: Bovine serum albumin; Cox-2: Cyclooxygenase 2; ECM: Extracellular matrix; FBS: Fetal bovine serum; IKK: IκB kinase; IL: Interleukin; LT-α: Lymphotoxin α; MMP: Matrix metalloproteinase; NF-κB: Nuclear factor κB; OA: Osteoarthritis; PARP: Poly(ADP-ribose) polymerase; PBS: Phosphate-buffered saline; RA: Rheumatoid arthritis; SO: Sense oligonucleotides; TNF: Tumor necrosis factor.

## Competing interests

The authors declare that they have no competing interests.

## Authors’ contributions

CB, PS and MS carried out all of the experiments and drafted the manuscript. BBA and MS were responsible for the experiment design and data analysis. CB, PS and MS participated in histological and immunohistochemical analyses. BBA and MS participated in the evaluation of each experiment. BBA, CB and MS revised the paper, provided technical support and drafted the final version of the manuscript. All authors read and approved the final manuscript.
